# A retrospective study of forensic cases of skin ulcerations in Danish pigs from 2000 to 2014

**DOI:** 10.1186/s13028-016-0229-0

**Published:** 2016-07-15

**Authors:** Kristiane Barington, Kristine Dich-Jørgensen, Henrik Elvang Jensen

**Affiliations:** Department of Veterinary Disease Biology, Faculty of Health and Medical Sciences, University of Copenhagen, Ridebanevej 3, DK-1870 Frederiksberg C, Denmark

**Keywords:** Forensic pathology, Pig, Ulcerations, Welfare

## Abstract

**Background:**

Ulcerations in pigs, as in other farm animals, are considered to be painful and therefore hampering the welfare. Farmers are obliged to provide an intervention to protect animals against unnecessary suffering and failure to do so is considered negligence. Moreover, animals with severe open wounds are considered unfit for transportation and so are pigs with ulcerations located on hernias. This paper presents a retrospective study of forensic case files concerning ulcerations in Danish pigs from 2000 to 2014. The aim of the study was to clarify the number of cases, the number of pigs, the anatomical localization and size of ulcerations, evaluate changes during years and the age of the lesions.

**Results:**

A total of 209 case files concerning 283 pigs with 459 ulcerations were included. In 2004, 2005, 2007–2009 and 2011, sows with shoulder ulcerations were the most frequently submitted, while in 2014 pigs with ulcerations on umbilical outpouchings dominated. The change in pattern on body location most likely reflects specific national regulations enforced from 2003 to 2009. The ulcerations were estimated to be from 4 h to several months old and the median diameter of ulcerations was 4 cm.

**Conclusions:**

Since 2004, the number of cases per year has declined. However, the number of affected pigs has remained almost constant from 2004 to 2014 (23.8 ± 8.5 pigs per year). The change in pattern on body parts with ulcerations likely reflected specific national regulations.

## Background

Ulcerations in pigs, as in other farm animals, are considered to be painful and therefore hampering the welfare. If neglected, an ulceration can result in a forensic case, i.e. being reported to the police [1, 2]. Most ulcerations are due to external trauma while others are due to fistulation to the skin surface from an underlying condition [3–7]. However, farmers are obliged to provide an intervention to protect animals against unnecessary suffering no matter the cause of the ulceration. Failure to do so is considered negligence and a violation of the European Union Council Directive concerning the protection of animals kept for farming purposes [8]. In addition, animals with severe open wounds are considered unfit for transportation [9]. It has been specified by the Danish Veterinary and Food Administration that animals with ulcerations larger than 3 cm in diameter are unsuitable for transportation from transit locations or for transport across borders [10]. Moreover, the Danish Animal Welfare Council has stated that transport of slaughter pigs with ulcerations located on hernias is prohibited [11, 12].

Since 2003, special attention has been drawn to shoulder ulcerations on sows in Denmark and other countries (e.g. England and Canada) [1, 2, 4, 13–15]. Consequently, the Danish Animal Welfare Council stated that all shoulder ulcerations involving the subcutaneous tissue or deeper structures are considered a violation of the Danish Animal Protection Act [16–19].

The aim of a veterinary forensic examination requested by the police is to perform a thorough and precise documentation of the lesions with respect to diagnosis, dimensions, anatomical localization, and an assessment of the age of the lesions [20, 21]. Depending on the case, the estimated age of ulcerations is used to interpret the degree of negligence; i.e., ulcerations left untreated for a longer period are legally considered more serious than acute lesions [18].

In Denmark, all veterinary forensic investigations are requested by the police and carried out at the University of Copenhagen. This paper presents a retrospective study of forensic case files concerning ulcerations in Danish pigs from 2000 to 2014 submitted to Department of Veterinary Disease Biology, Faculty of Health and Medical Sciences, University of Copenhagen. The aim of the study was to clarify the number of cases, the number of pigs, the anatomical localization and size of ulcerations, evaluate changes during years and the age of lesions.

## Methods

Case files concerning ulcerations in pigs sent for forensic investigation to the University of Copenhagen from 2000 to 2014 were examined retrospectively. A case file was defined as a single police record and included information regarding the number of pigs, the number and anatomical localizations of the ulcerations, the sex and age of the animal, the gross and histological descriptions of the ulcerations and usually photo documentation of lesions.

An ulceration was defined as a breach of the epidermis including the basal membrane [3]. Ulcerations included were due to an external force while case files concerning fistulation to the skin surface from underlying lesions were not included. All ulcerations were originally reported to the police by veterinary enforcement officers, i.e. by The Veterinary Task Force for livestock inspection, at transit locations, or at meat inspection at slaughterhouses.

The gross and histological evaluations of the ulcerations had been carried out by five experienced veterinary pathologist (i.e. professors with more than 10 years of experience with diagnostic pathology). Age estimations stated in the case files were made by the pathologists basing their estimations on the presence and amount of granulation tissue. In a few cases, in which granulation tissue was not grossly visible, the histological inflammatory reaction was used to state the age of the ulcerations. As reference to the estimation of age, studies of wound healing in pigs and humans were used [22–27]. Based on the gross (presence of granulation tissue) and histological descriptions, ulcerations were divided into four age intervals: (1) 0–3 days; (2) 4–7 days; (3) 8–28 days; and (4) >28 days. If granulation tissue was absent the ulceration was estimated to be less than 3 days old. In ulcerations with granulation tissue, the mean thickness was [mean ± standard deviation (SD)]: 4–7 days = 0.5 ± 0.3 cm; 8–28 days = 1.5 ± 1.2 cm; >28 days = 2.7 ± 2.5 cm. In ulcerations in which granulation tissue was not grossly visible, histology was applied to state the age of the ulceration. From these ulcerations new sections were cut and evaluated.

From the information given in the case files, the sex and age of the pigs were registered and pigs were grouped according to the anatomical localization of the ulceration: (1) shoulder, (2) umbilical outpouching (hernias, enterocystoma and preputial diverticula), (3) body (head, neck and back), (4) limb and (5) tail region. A pig with multiple ulcerations at different anatomical localizations was allocated to each of the specified anatomical localizations. For each of the anatomical localizations, measurements of the ulcerations were evaluated for normality and data were found to be nonparametric (SAS Institute 9.4). A Wilcoxon Rank Sum Test was used to compare the medians of the five groups (SAS Institute 9.4), and a *P* value below 0.05 was interpreted as a significant difference between groups.

## Results

**Fig. 1:**
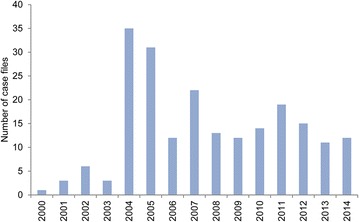
The annual number of forensic case files of skin ulcerations in pigs examined from 2000 to 2014

The inclusion criteria were fulfilled for 209 case files (Fig. 1). Each case file included 1–10 pigs with an average ($\bar{x}$) ± SD of 1.4 ± 1.2 pigs. Each pig had between 1 and 60 ulcerations (1.7 ± 3.7, $\bar{x}$ ± SD) with a median of 1. In total, tissue from 283 pigs had been submitted in which 459 ulcerations were described. In 40 of the 283 pigs, the exact number of ulcerations was not stated. The pigs were registered as females (53 %), males (5 %) and of unknown sex (42 %). Approximately half of the pigs were sows (51.2 %), while 34.3, 6.7, 0.4 and 7.4 % were slaughter pigs (5–6 months), younger pigs (<5 months), adult boars and pigs of unknown age, respectively.

**Fig. 2:**
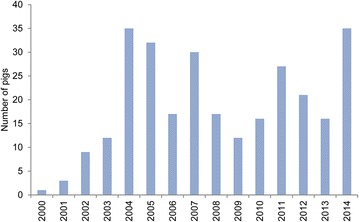
The annual number of pigs with skin ulcerations examined from 2000 to 2014

**Fig. 3:**
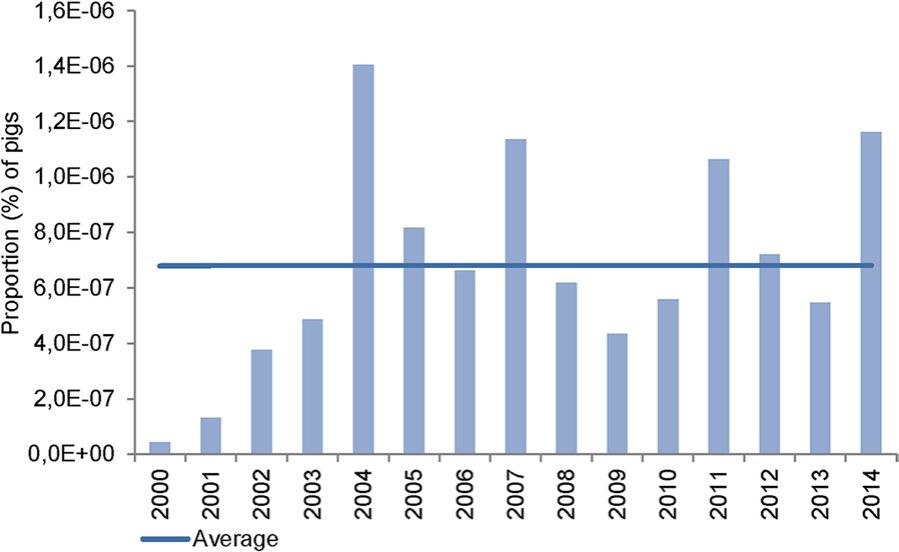
Proportion of pigs with skin ulcerations submitted for forensic examination in relation to the total number of pigs produced in Denmark annually from 2000 to 2014

**Fig. 4:**
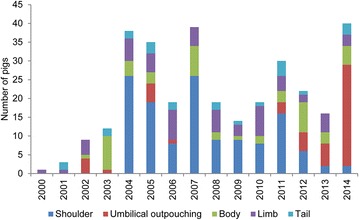
The annual number of pigs with skin ulcerations localized on the shoulder, umbilical outpouching, body, limbs or tail

During 2000–2003, 13 cases were sent for forensic investigation (Fig. 1). In 2004, the number of case files peaked (35 case files) being more than 11 times higher than in 2003 (3 case files). After 2004, the number of case files declined, however, the number of pigs with ulcerations each year showed an almost constant level of 23.5 ± 8.5 ($\bar{x}$ ± SD) pigs per year (Fig. 2). The proportion (%) of pigs submitted in relation to the number of produced pigs in Denmark showed roughly the same tendency as seen in Fig. 3. The pattern of distribution of ulcerations in the five groups of anatomical localization is presented in Fig. 4. In total, 131 out of 283 (46.3 %) of the pigs submitted from 2000 to 2014 were sows with shoulder ulcerations. In slaughter pigs and younger pigs, some ulcerations were located on abdominal outpouchings. In total, 52 out of 283 (18.4 %) of all pigs had an ulcerated outpouching in the abdominal region. Ulcerations on the body and limbs were present in pigs of all ages and were present in 49 out of 283 (17.3 %) and in 61 out of 283 (21.5 %) of pigs, respectively. Ulcerations in the tail region were present in 23 out of 283 pigs (8.1 %). The total sum of the percentages exceeds 100 % as 22 pigs had ulcerations located at more than one location.

In 90.8 % of the ulcerations (417 out of 459), measurements of dimensions were registered. The median diameter of all ulcerations was 4 cm. The median, minimum and maximum values for the diameter of ulcerations in each of the anatomical localizations and totally are presented in Table 1. Differences in the diameter of the ulcerations were highly dependent on the anatomical localization (*P* < 0.0001).

**Table 1:** The diameter of 417 skin ulcerations in forensic case files (2000–2014)

Anatomical localization	Number of ulcerations	Median diameter (cm)	Minimum diameter (cm)	Maximum diameter (cm)
Shoulder	159	5	1	11
Umbilical outpouching	56	5.8	0.7	21
Body	37	1	0.3	10
Limb	142	1.5	0.2	14
Tail	23	3	1	18
Total	417	4	0.2	21

The age had been determined for 65.4 % of the ulcerations (300 out of 459). The ulcerations were evaluated to be: (1) 0–3 days, n = 27; (2) 4–7 days, n = 3; (3) 8–28 days, n = 237; and (4) >28 days, n = 33.

New histological sections were cut in 29 out of 459 (6.3 %) ulcerations because granulation tissue had not been observed at gross evaluation. In a single ulceration, inflammation or granulation tissue was absent at the histological evaluation, and the age was stated as unknown. In another ulceration, in which the amount of granulation tissue could not be determined no estimate of an age was given. Three ulcerations were characterized by the infiltration of neutrophils and stated to be at least 4 h old. In nine ulcerations, both neutrophils and macrophages were present and the ulcerations were estimated to be between 6 and 24 h of age. In 15 ulcerations proliferation of fibroblasts was present and in some of the ulcerations newly formed capillaries were also observed. These ulcerations were stated to be 2–3 days old (n = 11) and between 16 and 32 h old (n = 4).

## Discussion

Despite the number of case files per year had declined since 2004, the number of pigs affected remained almost constant during the last 10 years suggesting fewer but more serious offenders (Figs. 1, 2). Since 2003, shoulder ulcerations have received increased attention as a reflection of the publication of several papers [1, 2, 4, 14] and statements from the Danish Animal Welfare Council on the subject [17–19]. The development in the number of sows with shoulder ulcerations submitted for forensic investigation reflects the increased attention on the subject (Fig. 4). The first paper addressing the problem with shoulder ulcerations in sows was published in 2003 [14] and already the year after more than 25 cases were received for forensic investigation (Fig. 4). It is also likely that the increase in pigs with ulcerations on umbilical outpouchings from 2011 to 2014 is a consequence of two specific regulations on transportation of animals with ulcerations on umbilical outpouchings enforced by the Danish Animal Welfare Council in 2008 and 2009 [11, 12]. In these regulations, it was deducted that pigs with umbilical outpouching with a diameter of more than 15 cm or with ulcerations were unfit for transportation. Before that time, statements on umbilical outpouching were vaguer; i.e. animals were unfit for transportation if they were hampered by the outpouching.

The median diameter (4 cm) of all ulcerations exceeded the threshold of 3 cm set by the Danish Veterinary and Food Administration regarding ulcerations being too large to allow transportation of the animal. The median diameter of ulcerations on shoulders and umbilical outpouchings both exceeded 3 cm, while the median diameter of ulcerations on the tail, body and limbs did not exceed 3 cm (Table 1).

Estimating the age of ulcerations is central to a forensic investigation, as this in a judicial setting will be used to interpret the degree of negligence or in apportioning blame. In the majority of ulcerations, the age was estimated based on the presence of granulation tissue at gross examination. In order to document the level of violence of animal protection legislations, it is crucial that all cases are examined and filed in a well-documented professional manner. Therefore, in Denmark all requests for forensic examination of porcine ulcerations are taken care of at the University of Copenhagen. Moreover, the filing of cases is also highly important when sending out information material to pig producers, arrangement of courses and communicating with the authorities, e.g. the police and The Danish Welfare Council.

## Conclusions

Since 2004, the number of cases per year has declined while the number of affected pigs has remained almost constant. The changes in anatomical localization of the ulcerations during 2000–2014 likely reflected specific national regulations.
